# Assessing scale-dependency of climate risks in coffee-based agroforestry systems

**DOI:** 10.1038/s41598-024-58790-5

**Published:** 2024-04-05

**Authors:** Vivekananda M. Byrareddy, Jarrod Kath, Louis Kouadio, Shahbaz Mushtaq, Vellingiri Geethalakshmi

**Affiliations:** 1 Centre for Applied Climate Sciences, Institute for Life Sciences and the Environment, University of Southern Queensland, Toowoomba, QLD 4350 Australia; 2 SQNNSW Drought Resilience Adoption and Innovation Hub, Institute for Life Sciences and the Environment, University of Southern Queensland, Toowoomba, QLD 4350 Australia; 3 Faculty of Health, Engineering and Sciences, School of Agriculture and Environmental Science, University of Southern Queensland, Toowoomba, QLD 4350 Australia; 4 Tamil Nadu Agricultural University, Coimbatore, Tamil Nadu 641003 India

**Keywords:** *Coffea canephora*, Agroforestry, Climate variability, Climate change, Adaptive management, Plant development, Plant stress responses, Climate change

## Abstract

Agroforestry is a management strategy for mitigating the negative impacts of climate and adapting to sustainable farming systems. The successful implementation of agroforestry strategies requires that climate risks are appropriately assessed. The spatial scale, a critical determinant influencing climate impact assessments and, subsequently, agroforestry strategies, has been an overlooked dimension in the literature. In this study, climate risk impacts on robusta coffee production were investigated at different spatial scales in coffee-based agroforestry systems across India. Data from 314 coffee farms distributed across the districts of Chikmagalur and Coorg (Karnataka state) and Wayanad (Kerala state) were collected during the 2015/2016 to 2017/2018 coffee seasons and were used to quantify the key climate drivers of coffee yield. Projected climate data for two scenarios of change in global climate corresponding to (1) current baseline conditions (1985–2015) and (2) global mean temperatures 2 °C above preindustrial levels were then used to assess impacts on robusta coffee yield. Results indicated that at the district scale rainfall variability predominantly constrained coffee productivity, while at a broader regional scale, maximum temperature was the most important factor. Under a 2 °C global warming scenario relative to the baseline (1985–2015) climatic conditions, the changes in coffee yield exhibited spatial-scale dependent disparities. Whilst modest increases in yield (up to 5%) were projected from district-scale models, at the regional scale, reductions in coffee yield by 10–20% on average were found. These divergent impacts of climate risks underscore the imperative for coffee-based agroforestry systems to develop strategies that operate effectively at various scales to ensure better resilience to the changing climate.

## Introduction

Coffee is among the top traded agricultural commodities in the world^1^. Climate variability and change are increasingly threatening the profitability and sustainability of the coffee industry^2–6^. Prolonged droughts and high temperatures during the crop season, in combination with heavy rains and frosts, not only affect blossoming and fruit setting but also cherry development and filling, thereby reducing coffee bean yield and/or decreasing bean quality^4,7–10^. With longer and more intense droughts coupled with higher temperatures increasing across most of the world’s coffee growing areas^11,12^, strategies such as relocation of coffee farming, adapting coffee farming practices and/or the development of new coffee crops are being developed to ensure the sustainability and profitability of coffee production in the future^13,14^.

Agroforestry is a potential management strategy for mitigating climate variability and change impacts in coffee farming systems^13,15–17^. Coffee-based agroforestry systems (CAFS) consist of coffee intercropped with a diverse canopy of native or nonnative forest trees in high to moderate shade, with varying shade cover management. There are various benefits of CAFS, including improved soil chemical and physical properties, the creation of a microclimate at the farm level that can lower soil and air temperatures and acts as a buffer against extreme temperature fluctuations within the farm, and an increase in ecosystem productivity per area^13,15,18,19^. CAFS can also provide additional income to farmers^20,21^. However, similar to any other ecosystem, CAFS are vulnerable to large-scale annual and multi-year fluctuations in weather and climate. In countries that manage diverse shade trees in all or parts of their coffee-producing regions (i.e., India, Colombia, Haiti)^22^, the vulnerability of these managed ecosystems to climate is expected to increase over the coming decades^23–25^. The appropriate scale at which to describe the impact of climate on coffee under the scope of varying CAFS set-ups needs, therefore, explicit investigation.

When assessing climate risks and climate change impacts on coffee, studies have mostly focused on either the suitability of land areas for coffee production^2,16,26^ or the assessment of potential coffee yield under projected climate conditions^4,27,28^, the exposure and vulnerability of coffee-producing regions to changes in climate hazards^29^ or the proportion of variation in coffee yield explained by climate predictors (i.e., rainfall and temperature)^30,31^. However, the risks associated with climate variability at different spatial scales in CAFS under various management practices have yet to be fully investigated.

At different scales, climate risks such as excess or deficit rainfall, dry spells or frosts, may differ depending on the topographical characteristics and the management practices that dominate the CAFS. If climate impacts vary considerably between scales, i.e., the particular climate driver(s) most important for yield vary, then extrapolating findings from global and country-level studies^2,4,9,31^ may have limited utility for informing smaller farm-scale climate adaptation responses. Likewise, smaller experimental and farm-scale studies^32^ may not be relevant for understanding the larger scale regional and global impacts of climate change. Understanding the impact of climate at different spatial scales is therefore crucial for accurately assessing the threat current and future climate variability pose to CAFS.

In this study, climate risk impacts on robusta coffee (*Coffea canephora* Pierre ex A. Froehner) yield at different spatial scales in CAFS were investigated for the key robusta coffee-growing regions in India, the third largest Asian robusta coffee-producing country^33^. Robusta coffee production in India accounts for approximately 70% of the national production, for an average annual cultivated land area estimated ca. 237,000 ha (that is, approximately 50% of the national coffee acreage)^34^.

In India, coffee is grown under native and nonnative shade tree cover and presents a range of management practices across different landscapes^21,35^, making it an ideal system for investigating climate impacts at different scales. In the study we hypothesized that climate risks and their impact on coffee yield would vary across spatial scales, i.e., within and across regions. Therefore, mitigation and adaptation strategies must be designed and implemented accordingly. Agroforestry is pivotal to the success and sustainability of Indian coffee farming systems since it helps protect against the adverse effects of prolonged dry spells and hot summers, particularly during the dry season (November–March)^6,21^. The findings of this study could inform and support spatial planning and actions for improved climate risk management at multiple spatial scales in robusta CAFS in India and other coffee-producing countries around the world.

## Methods

### Study area

**Figure 1:**
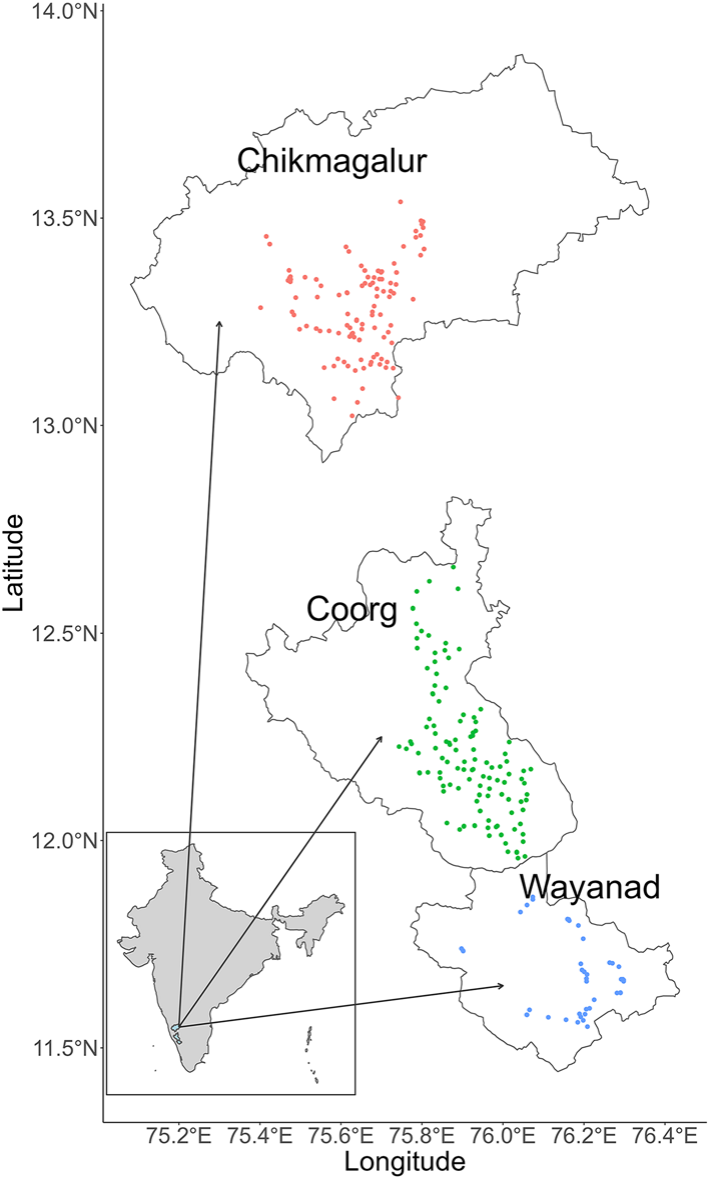
The study regions and spatial distribution of the surveyed robusta coffee farms across the districts of Chikmagalur, Coorg (Karnataka state) and Wayanad (Kerala state) in India. The total number of surveyed farmers each year was 122, 146, and 46 in Chikmagalur, Coorg, and Wayanad, respectively.

Data were collected from 314 coffee farmers across the districts of Chikmagalur, Coorg (Karnataka state), and Wayanad (Kerala state) in India (Fig. 1) during the 2015/2016 to 2017/2018 coffee seasons. Karnataka and Kerala are the main robusta coffee-producing states in India and account for 93% of national production^34^. The robusta coffee calendar in the study regions can be roughly divided into four periods: the January to April period, during which flower-bud initiation, blossoming, and fruit setting occur; the May to October period, encompassing cherry development and maturation; the October to December period, encompassing ripening and harvest; and the dormancy stage from December to January^36^. In Wayanad, coffee is grown in rainfed conditions, whereas in Chikmagalur and Coorg supplemental deficit irrigation is adopted, namely, to trigger flowering when blossom showers (occurring generally during March–April) are not sufficient. Robusta coffee flowering in the study regions usually falls during the dry season and coincides with the summer months^30^, with backing showers needed within 20–25 days of first blossom showers to allow for good fruit setting^36^.

### Farm data

Farm level data were collected using designed questionnaires. The sampling was performed to represent the range of farm sizes and crop management practices. Coffee farmers kept their farm activity records using bookkeeping, which is also valuable for coffee certification and traceability programmes. The surveyed farmers were selected so as to represent the range of climate, the proportion of coffee growing area, use of irrigation water, and farming systems. The questionnaire was reviewed and approved by the ethical committee of ECOM Agroindustrial Corp. Ltd. Data collection was carried out in accordance with relevant guidelines and regulations. Informed consent was obtained from all participants before the interview.

The total number of farmers surveyed each year was 122, 146, and 46 in Chikmagalur, Coorg, and Wayanad, respectively; that is 942 observational data records for the three coffee seasons. Data collected included farm characteristics (farm size, type of shade trees—native or exotic tree species), crop management practices (plant density, fertilizer types and rates, pest and disease management strategies, irrigation water use), and production data (i.e., annual coffee bean yield). The surveyed coffee farms were composed of robusta coffee and native tree species, with tree spacings of 3 m × 3 m and 12 m × 14 m for coffee and shade trees, respectively^36^. All the data were anonymised before any analysis was performed in this study.

### Climate data

Monthly climate data for the period 1985–2018, sourced from the TerraClimate dataset (~ 4 km resolution)^37,38^, were used. Climate projections for the study area were for two levels of change in global climate corresponding to (1) baseline conditions representing those from 1985 to 2015 and (2) a global mean temperature 2 °C above the preindustrial period (1850–1879)^37,38^. The approach from Refs.^37,38^ for developing future climate projections uses monthly data from 23 Coupled Model Intercomparison Project 5 (CMIP5) climate models and observational records to scale changes in individual climate variables^38^. Details of the pattern scaling approach, as well as information about the 23 different Coupled Model Intercomparison Project 5 (CMIP5) climate models used, are provided in Ref.^38^.

**Figure 2:**
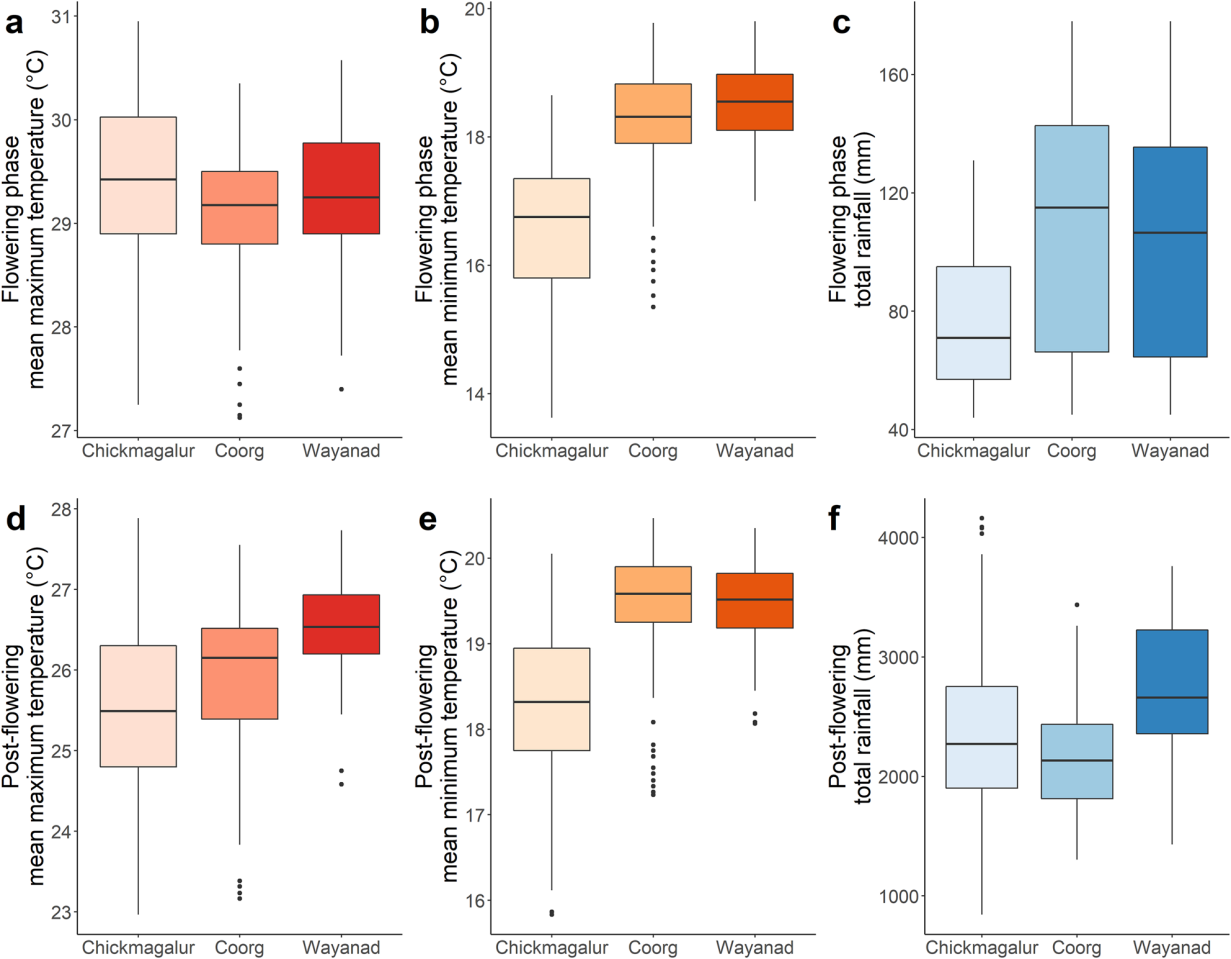
Distribution of mean maximum and minimum temperatures, and total rainfall across the Chikmagalur, Coorg, and Wayanad districts in India for the period 2015–2018. (**a**–**c**): values during the flowering period (January–April); (**d**–**f**): values during the post-flowering period (May to August). In a boxplot, the top and bottom of the box represent the 75th and 25th percentiles, respectively; the solid line indicates the median. The whiskers on the top and bottom represent the largest and smallest values within the 1.5 times interquartile range above the 3rd and 1st quantiles, respectively. Black points are outliers.

In this study, we focused on the flowering (January–April) and post-flowering (May–August) periods as this is when coffee production is most sensitive to climatic variability^4,8^. For each farm, the total rainfall, mean, minimum and maximum temperatures were computed each year for the flowering and post-flowering periods. Variations in each of the climate predictors for the period 2015–2018 are presented in Fig. 2.

### Data analysis

Climate impacts were assessed at two different scales: (1) regional, encompassing the entire study region and including all sample farms, and (2) district, covering the three distinct districts of Chikmagalur, Coorg and Wayanad (Fig. 1). All data analyses were carried out in R^39^.

### Identifying the key climate drivers of robusta coffee production

A generalized additive regression model (GAM)^40,41^ and multimodel selection^42^ were used to identify the key climate drivers of coffee yield. In the GAM coffee yield was modeled as a nonlinear function of climate variables for each site and year using a scaled t distribution to account for the heavy tailed distribution of yield data. A random effect for each site and year was included to account for the repeat measurements for each year at the district level. Random effects control for non‐independence by constraining non‐independent observations to have the same intercept^43^. For example, yield observations from a particular district, may be more similar (e.g., higher on average if soils and management techniques are better) relative to yield observations from other areas. There were six potential climate predictors (maximum temperature, minimum temperature, and total rainfall for both the flowering and post-flowering periods) in the global model.

To select the best climate predictor(s) for each of the periods (flowering and post-flowering) the second-order Akaike Information Criterion (AICc)-based model selection^42^ was used. This approach tests all possible model combinations of predictors and provides an AICc for these, which is used to rank model performance or parsimony. AICc is a stricter form of AIC and accounts well for situations when there are limited sample sizes^42^, as occurs here for our smaller-scale analyses. As with AIC, lower AICc values indicate better model performance and model parsimony. From model selection based on AICc, the most parsimonious model, that is, the one with the lowest AICc was selected. Model selection also accounted for collinearity, with no predictors with a Pearson coefficient of correlation > 0.7 being included in any of the models tested^44^. AICc-based model selection was performed using the ‘*MuMIn*’ package^45^.

### Assessing the impacts of climate change scenarios on robusta coffee yield across different spatial scales

The best models selected for the regional scale (all sample farms within the Karnataka and Kerala states) and district scale (sample farms for each of the three districts Chikmagalur, Coorg, and Wayanad) were used to assess the percentage change in robusta coffee yield across the study area in the future under two different scenarios corresponding to (1) baseline conditions (1985–2015) and (2) projections under a global mean temperature scenario of 2 °C. Under each scenario, we projected a percentage change in yield based on the best model for each district and the regional model, which encompassed all districts.

## Results

### Key climate drivers of robusta coffee yield variability across scales

The key drivers of robusta coffee yields in the CAFS we assessed across India varied considerably (Tables 1 and 2). At the regional scale encompassing our entire study region (Karnataka and Kerala states), post-flowering maximum temperatures were the most important climate driver of coffee yields (Table 1). In contrast, at the smaller district scale in Wayanad and Coorg, only rainfall-related variables were selected in the best models for explaining variation in coffee yields (Table 1).

**Table 1:** Model selection results (showing all models < delta 2) at the district level and across all regions combined.

Region	Flowering phase			Post-flowering phase			Df	LogLik^a^	AICc^b^	Delta	Weight
	Tmax	Tmin	Rain	Tmax	Tmin	Rain					
Wayanad	−	−	** + **	−	−	** + **	44	24.33	89.36	0	0.29
Coorg	−	−	** + **	−	−	−	152	84.83	298.03	0	0.13
Chikmagalur	−	−	−	** + **	−	−	126	203.99	−21.95	0	0.09
All regions	−	−	−	** + **	−	−	310	292.96	345.18	0	0.22

^a^Log Likelihood.

^b^Second-order Akaike Information Criterion.

**Table 2:** Statistical indicators of the best selected models at the combined regional and district levels.

Region	Predictor	*p* value^a^	Adjusted R^2^	Deviance (%)	N
Wayanad	Flowering-rain	0.0495*	0.578	65.6	129
	Post-flowering-rain	0.0071**			
Coorg	Flowering-rain	0.0033**	0.829	87.5	438
Chickmagalur	Post-flowering-Tmax	0.4956	0.907	83	366
All regions	Post-flowering-Tmax	0.0023**	0.864	90.2	933

^a^Statistical significance: *p < 0.05, **p < 0.01, ***p < 0.001.

### Regional-scale yield variation is best explained by post-flowering season temperature variation

**Figure 3:**
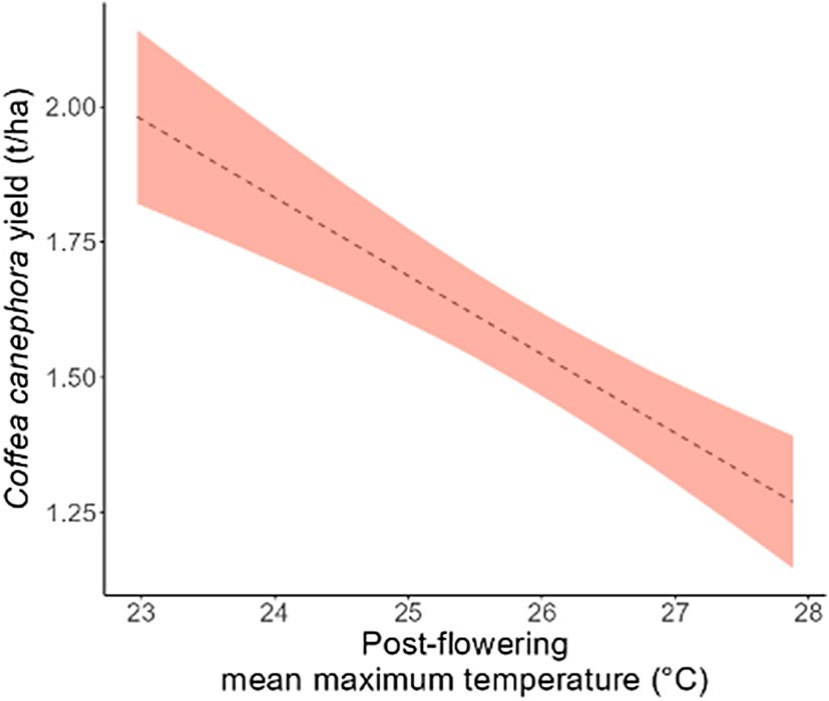
The relationship between post-flowering mean maximum temperature and robusta coffee yield at the regional level (across all farms assessed in the study). The shaded area is the 95% confidence interval from the model predictions.

At the regional scale, higher mean maximum temperatures were linearly and negatively related to yields (Fig. 3). In the post-flowering season, yields for mean maximum temperature of 28 °C were predicted to be approximately 700 kg/ha lower than those for a mean maximum temperature of 23 °C. Each degree increase in mean maximum temperatures, therefore, corresponded to an approximately 140 kg/ha decline in coffee yield (Fig. 3).

### District-level yields are best explained by rainfall

**Figure 4:**
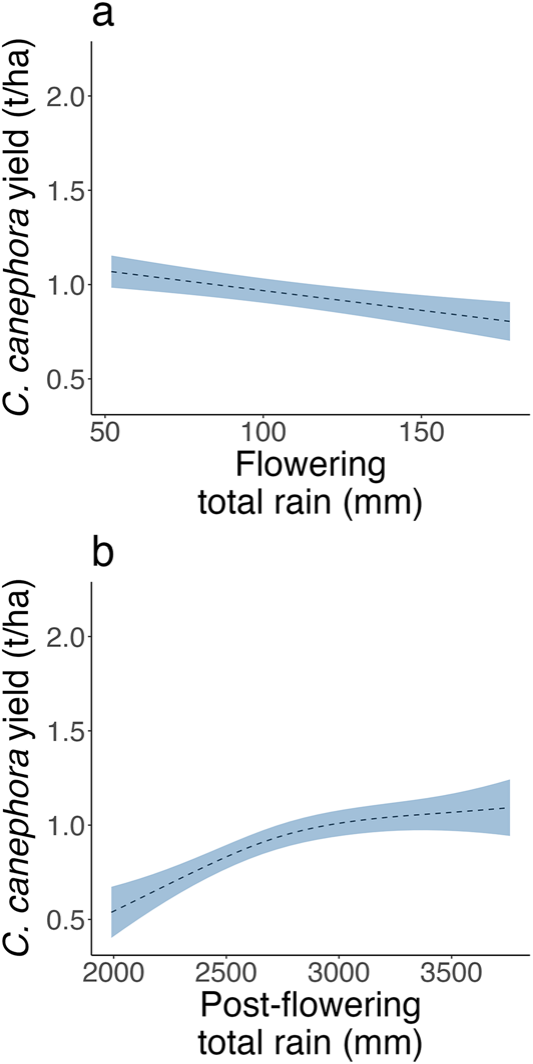
Relationships between robusta coffee yield and key climate drivers identified through model selection at the district level (see Table 1) for (**a**) flowering rainfall and (**b**) post-flowering rainfall in Wayanad. The shaded area in the figure represents the 95% confidence interval from the model predictions.

In contrast to the regional scale models, district-level yields were best explained by variations in rainfall (Fig. 4). In Wayanad, rainfall was a key predictor of yield variation in both the flowering and post-flowering periods (Fig. 4a, b). The relationship between rainfall and robusta coffee yields was negative during flowering; that is, higher rainfall corresponded to lower coffee yields (Fig. 4a). The opposite (higher rainfall corresponded to higher coffee yields) was found for the post-flowering period. During the post-flowering period in Wayanad, there was also a notable nonlinear relationship between rainfall and coffee yield, such that yields did not begin to decline until rainfall fell below 3000 mm (Fig. 4b). Once rainfall fell below 3000 mm, yields declined strongly, by approximately 400 kg/ha as rainfall declined to 2000 mm (Fig. 4b).

**Figure 5:**
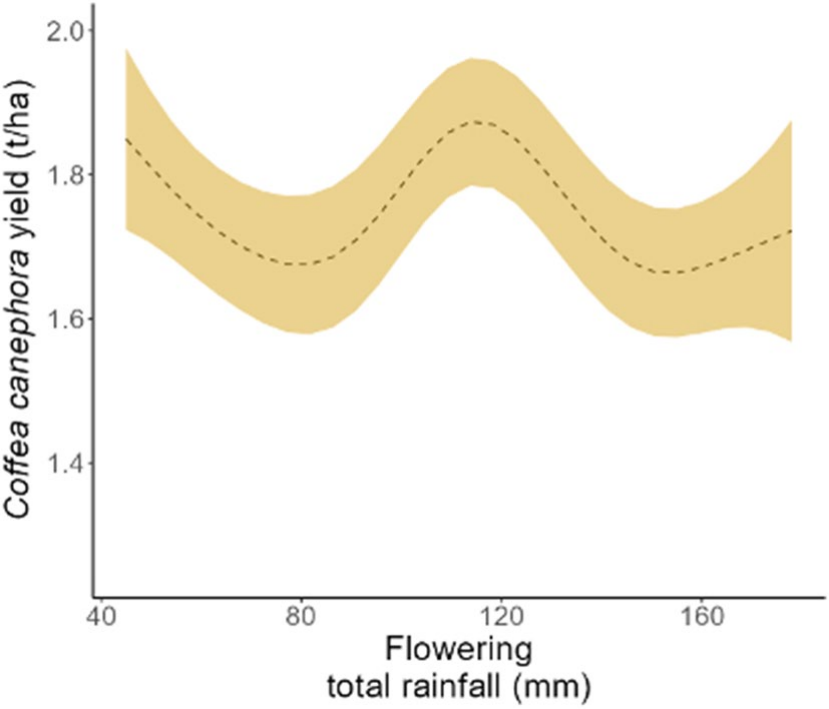
The relationship between the key climate drivers of robusta yield at the district level identified in Coorg. The shaded area in the figure represents the 95% confidence interval from the model predictions.

In Coorg, rainfall was also important, but only during the flowering period. There was a distinct inverted ‘U’ relationship between flowering total rainfall and coffee yield for that district (Fig. 5). Increasing rainfall from 80 to 100 mm corresponded to increased coffee yield, at which point the yield plateaued, and further rainfall (rainfall above 100 mm) was predicted to decrease yields. The lowest coffee yields (~ 1500 kg/ha) were predicted to occur at the highest flowering period rainfall observations (> 160 mm) (Fig. 5). In Chikmagalur, maximum temperature was the only variable identified in the best model, but its relationship with robusta coffee yield was not statistically significant (*p* > 0.05; Table 2).

### Impacts of climate change scenarios on robusta coffee yield across different spatial scales

**Figure 6:**
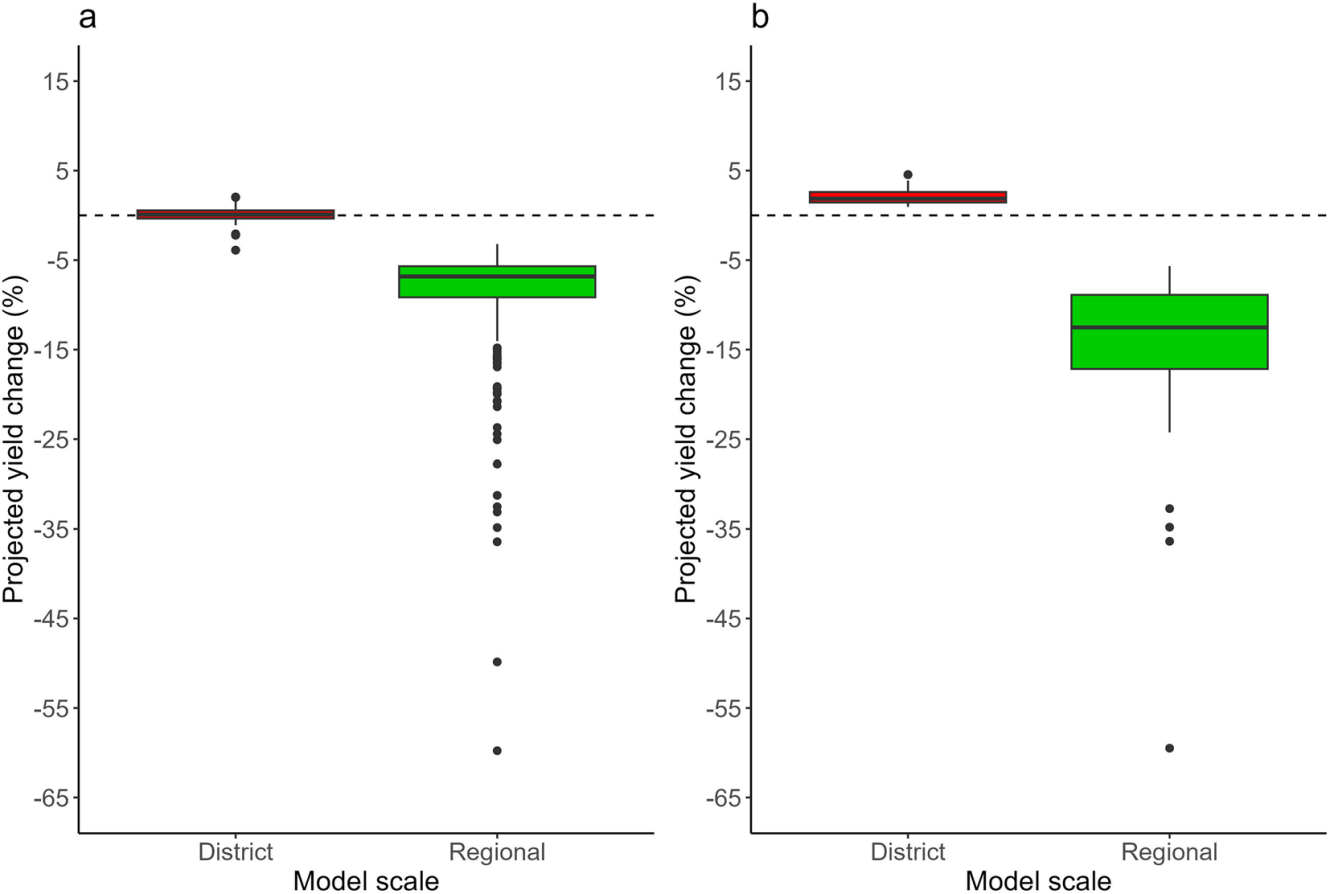
Boxplots showing the distribution of projected percentage changes in robusta coffee yield under a 2 °C global warming relative to baseline (1985–2015) climatic conditions for the best model at the district level and regional level for (**a**) Coorg and (**b**) Wayanad. No results are shown for Chickmagalur as there were no climate variables significantly related to yield at the district level for this area (*p* > 0.05; Table 2). In the boxplots, the top and bottom of the box represent the 75th and 25th percentiles, respectively; the solid line indicates the median. The whiskers on the top and bottom represent the largest and smallest values within the 1.5 times interquartile range above the 3rd and 1st quantiles, respectively. Black points are outliers.

The projected impacts of climate change on robusta coffee yields vary depending on whether district-level or regional-level models are utilised. At the district level where rainfall was identified as the key limiting factor of robusta coffee yields, virtually no impact on yields is anticipated in Coorg under a 2 °C warming scenario (Fig. 6a). In contrast, at Wayanad, slight positive changes (up to 5%) were found under a 2 °C warming scenario relative to the baseline (1985–2015) climatic conditions (Fig. 6b). In contrast, when projecting from the regional scale model, for which post-flowering maximum temperature was identified as the key limiting factor on production, the results showed decreases in robusta coffee yields under a 2 °C warming scenario for both Coorg and Waynad (Fig. 6). The median changes were − 7% and − 12% for Coorg and Waynad, respectively, with higher decreases found in the latter district (Fig. 6b).

**Figure 7:**
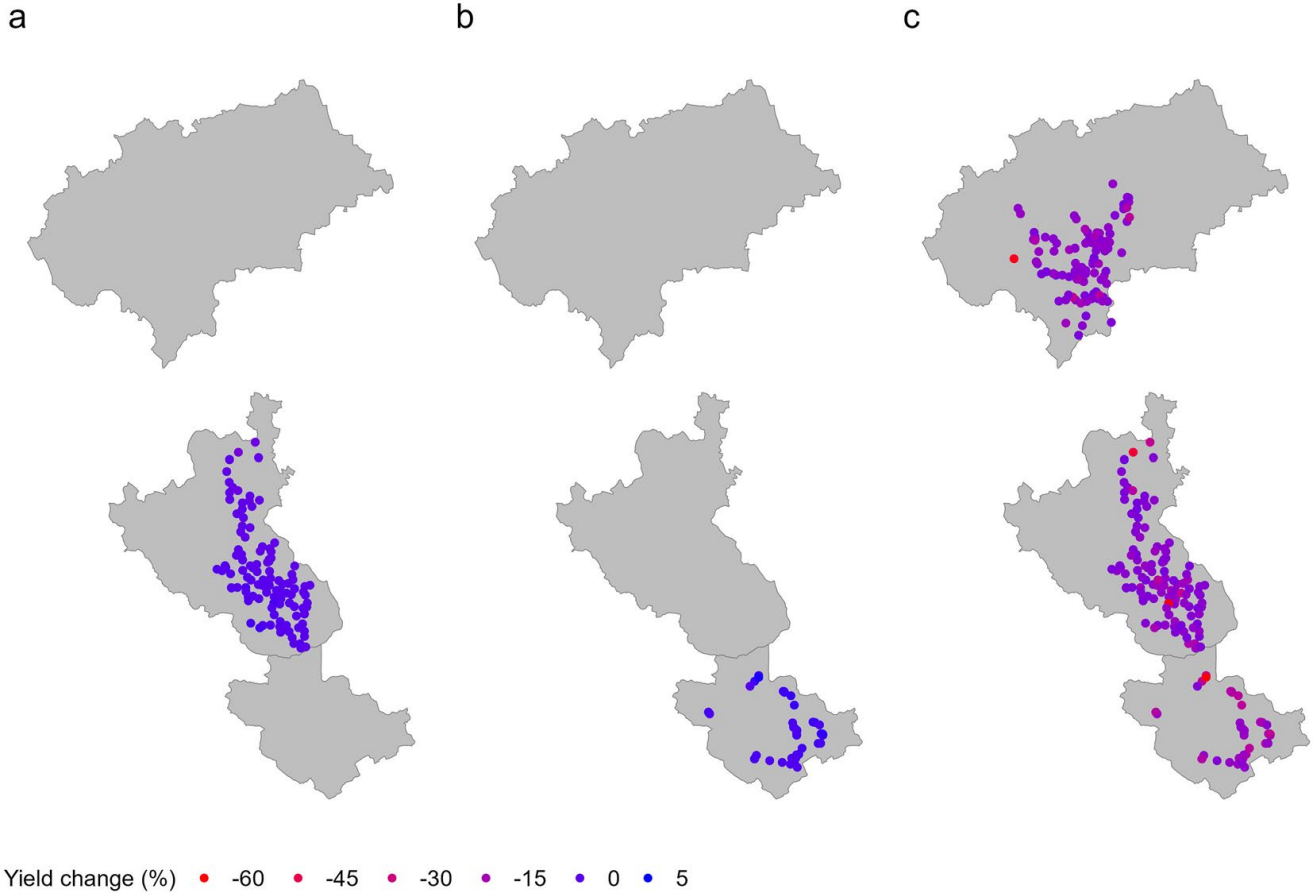
The spatial distribution of the projected mean change (%) in robusta coffee yield for each farm under a 2 °C global warming scenario relative to baseline (1985–2015) climatic conditions for the best model at the district scale in (**a**) Coorg and (**b**) Wayanad, and (**c**) the regional scale (all data pooled). Note that for the district-level model, no results are shown for Chickmagalur, as there were no climate variables significantly related to yield for this area (*p* > 0.05; Table 2).

The spatial distribution of the projected changes in robusta coffee yields at the district and regional scales is presented in Fig. 7. Irrespective of the scale, there were no distinct spatial patterns across the study districts. For instance, when using the regional-level model for Coorg, projected decreases of 15% and 30% were found for multiple farms within the same vicinity (Fig. 7).

## Discussion

The risks associated with climate variability and change during the flowering and post-flowering periods on robusta coffee production at different spatial scales were investigated. The results showed varying scale-dependent relationships between robusta coffee yield and flowering and post-flowering rainfall and temperature variables in the study areas. At the regional scale (pooled data for all three districts), post-flowering maximum temperature was selected as the best variable explaining the variability in robusta coffee yield, with lower maximum temperatures being associated with higher yields. These results concur with previous findings indicating that high temperatures adversely impact robusta coffee yield^7,31,46,47^. Bud development and berry filling, two key yield determinants in coffee yield, can be drastically and negatively affected under high temperature conditions^7,46^, which may explain the relationships observed in this study.

At the district scale (i.e., in coffee farms within each district), variations in rainfall during flowering mostly explained the variability in robusta CAFS across the study area. Negative impacts of relatively higher rainfall during flowering on robusta coffee yield were found across Coorg and Wayanad. Such results are in line with other work^46,47^. This could be related to various factors including reduced pollination potential and abortion of flowers in the case of intense rainfall early and late flowering, respectively, which both result in lower overall fruit set and low final yield^10,15^. On the other hand, good rainfall conditions during the post-flowering period yielded better robusta coffee productivity, namely, across Wayanad, where robusta coffee is typically grown under rainfed conditions. Good rainfall conditions are most often beneficial to cherry development in robusta coffee^8,15^, although such conditions can be conducive to increased pest and disease infestations, which in turn may lead to yield loss if not well managed^46,47^. The selection of post-flowering maximum temperature as the variable explaining the most variability in robusta coffee yield in Chikmagalur suggests that the effects of localised microclimatic conditions on coffee yields were also identified through the model in our study. Indeed, the presence of a shade canopy can buffer extreme temperatures, reduce soil evaporation and crop transpiration, and improve production^15,48,49^.

To cope with the adverse impacts of climate variability on coffee productivity in CAFS a range of management strategies exist, including reliance on supplemental deficit irrigation, infrastructural investments in farms to enhance rainwater storage capability, tailored shade management to avoid competition for light during critical coffee phenological stages, diversification and careful selection of shade tree species (preferably context-specific species with rooting systems that would avoid competition for water and nutrients), crop and income diversifications, and adoption of crop insurance schemes^16,21,50,51^.

Our study shows that under a 2 °C warning scenario relative to baseline (1985–2015) climatic conditions, the projected coffee yield changes varied between + 5 and − 20% depending on the scale, with reductions found at the regional scale (i.e., projections made using the regional-scale model). With increasing interest in adopting CAFS as a strategy to reduce the vulnerability of both farmers and the global coffee sector to climate change^13,16,52^, as well as to enhance ecosystem services in coffee-growing regions^53,54^, it is important to identify the risks associated with climate variability in CAFS at different spatial scales for tailored and improved risk management. To date, studies have focused on the analysis of the co-benefits, synergies, and trade-offs of shade canopy and robusta coffee productivity in CAFS, with little attention given to scale-dependent climate risk levels^14,49^. Our study highlights the need to consider differentiated responses based on the main climate driver and its scale-dependent level of risk.

At finer spatial scales, mitigation and adaptation strategies should focus on helping farmers improve their shade management (pruning method and intensity, choice of context-specific and multipurpose tree species based on farmer knowledge), adopt practices that would increase water and nutrient use efficiencies, and use drought- and heat-stress-adapted varieties. Given that yield potentials and responses to environmental stresses in robusta coffee are genotype- and location-specific^15,55^, the adoption of new robusta coffee varieties needs to be preceded by appropriate pilot phases and good communications of their outcomes within farmers for better uptake.

At coarser spatial scales, land management and use that minimize the effects of regional warnings should be prioritized. This would require long-term institutional arrangements among multiple stakeholders, and multisectoral planning and implementation of appropriate adaptation actions to avoid risks of maladaptation^56,57^. Although farm relocation to suitable cropping areas may be seen as a potential climate adaptation strategy^58–60^, it is critical to investigate the feasibility of such strategies according to local contexts (e.g., suitable sites located in protected areas, prevailing land policies). Moreover, relocating farms will require adequate financial, technical, and social resources, which in most cases are unaffordable for smallholder coffee farmers, unless they are subsidised.

There are some limitations in this study that indicate a need for further research. Climatic variables such as solar radiation, wind speed, vapor pressure deficit, and soil moisture conditions were not included, but their effects may also vary across scales. As these factors could drive robusta coffee productivity^13,55,61^, considering the potential interactions of these factors with robusta coffee productivity would provide further insights into the climate risk levels in CAFS and help improve risk management. Moreover, targeted data collection and quantitative analysis should be undertaken in future research to explore the potential impacts of climate variability and change on shade trees in CAFS and understand how interspecific interactions will respond to climate change^62,63^. This would help improve current adaptive management strategies for CAFS and ensure better preparedness for future climate conditions for profitable and sustainable robusta coffee farming systems.

In summary, the study highlights the need to consider differentiated responses based on the main climate driver and its scale-dependent level of risk in coffee-based agroforestry systems. Such findings could inform and support spatial planning and actions for improved climate risk management at multiple spatial scales in robusta coffee-based agroforestry systems in India and other coffee-producing countries around the world.

## Data Availability

The datasets generated during and/or analysed during the current study are not publicly available due to privacy concerns but are available from the corresponding author on reasonable request.

## References

[1] FAOSTAT. (2021).

[2] Bunn C, Läderach P, Ovalle Rivera O, Kirschke D (2015). A bitter cup: Climate change profile of global production of Arabica and Robusta coffee. Clim. Chang..

[3] Schroth G, Läderach P, Blackburn Cuero DS, Neilson J, Bunn C (2015). Winner or loser of climate change? A modeling study of current and future climatic suitability of Arabica coffee in Indonesia. Reg. Environ. Chang..

[4] Craparo ACW, Van Asten PJA, Läderach P, Jassogne LTP, Grab SW (2015). Coffea arabica yields decline in Tanzania due to climate change: Global implications. Agric. For. Meteorol..

[5] Watts, C. & The Climate Institute. A brewing storm: The climate change risks to coffee. The Climate Institute, Australia. https://files.fairtrade.net/publications/2016_TCI_ABrewingStorm.pdf (2016).

[6] Jayakumar M, Rajavel M, Surendran U, Gopinath G, Ramamoorthy K (2017). Impact of climate variability on coffee yield in India—With a micro-level case study using long-term coffee yield data of humid tropical Kerala. Clim. Chang..

[7] DaMatta FM, Ramalho JDC (2006). Impacts of drought and temperature stress on coffee physiology and production: A review. Braz. J. Plant Physiol..

[8] Kath J, Mittahalli Byrareddy V, Mushtaq S, Craparo A, Porcel M (2021). Temperature and rainfall impacts on robusta coffee bean characteristics. Clim. Risk Manag..

[9] Kath J (2022). Vapour pressure deficit determines critical thresholds for global coffee production under climate change. Nat. Food.

[10] Kath J, Byrareddy VM, Reardon-Smith K, Mushtaq S (2023). Early flowering changes robusta coffee yield responses to climate stress and management. Sci. Tot. Environ..

[11] IPCC. *Climate change 2021: The physical science basis. Contribution of Working Group I to the Sixth Assessment Report of the Intergovernmental Panel on Climate Change*. (Cambridge University Press, 2021).

[12] Richardson D (2023). Synchronous climate hazards pose an increasing challenge to global coffee production. PLOS Clim..

[13] Lin BB (2007). Agroforestry management as an adaptive strategy against potential microclimate extremes in coffee agriculture. Agric. For. Meteorol..

[14] Koutouleas A (2022). Shaded-coffee: A nature-based strategy for coffee production under climate change? A review. Front. Sustain. Food Syst..

[15] DaMatta FM (2004). Ecophysiological constraints on the production of shaded and unshaded coffee: A review. Field Crops Res..

[16] Gomes LC (2020). Agroforestry systems can mitigate the impacts of climate change on coffee production: A spatially explicit assessment in Brazil. Agric. Ecosyst. Environ..

[17] Harvey CA (2018). Climate change impacts and adaptation among smallholder farmers in Central America. Agric. Food Secur..

[18] Bhagwat SA, Kushalappa CG, Williams PH, Brown ND (2005). A landscape approach to biodiversity conservation of sacred groves in the Western Ghats of India. Conserv. Biol..

[19] Meylan L (2017). Evaluating the effect of shade trees on provision of ecosystem services in intensively managed coffee plantations. Agric. Ecosyst. Environ..

[20] Jezeer RE, Verweij PA, Santos MJ, Boot RGA (2017). Shaded coffee and cocoa—Double dividend for biodiversity and small-scale farmers. Ecol. Econ..

[21] Nesper M, Kueffer C, Krishnan S, Kushalappa CG, Ghazoul J (2017). Shade tree diversity enhances coffee production and quality in agroforestry systems in the Western Ghats. Agric. Ecosyst. Environ..

[22] Jha S (2014). Shade coffee: Update on a disappearing refuge for biodiversity. BioScience.

[23] García L JC, Posada-Suárez H, Läderach P (2014). Recommendations for the regionalizing of coffee cultivation in Colombia: A methodological proposal based on agro-climatic indices. PLOS ONE.

[24] Chengappa PG, Devika CM (2016). Climate variability concerns for the future of coffee in India: An exploratory study. Int. J. Environ. Agric. Biotechnol..

[25] de Sousa K, van Zonneveld M, Holmgren M, Kindt R, Ordoñez JC (2019). The future of coffee and cocoa agroforestry in a warmer Mesoamerica. Sci. Rep..

[26] Ovalle-Rivera O, Läderach P, Bunn C, Obersteiner M, Schroth G (2015). Projected shifts in *Coffea arabica* suitability among major global producing regions due to climate change. PLOS ONE.

[27] Zullo J, Pinto HS, Eduardo Delgado A (2006). Impact assessment study of climate change on agricultural zoning. Met. Appl..

[28] Tavares PDS, Giarolla A, Chou SC, Silva AJDP, Lyra ADA (2018). Climate change impact on the potential yield of Arabica coffee in southeast Brazil. Reg. Environ. Chang..

[29] Koh I, Garrett R, Janetos A, Mueller ND (2020). Climate risks to Brazilian coffee production. Environ. Res. Lett..

[30] Jayakumar M, Rajavel M, Surendran U (2016). Climate-based statistical regression models for crop yield forecasting of coffee in humid tropical Kerala, India. Int. J. Biometeorol..

[31] Kath J (2020). Not so robust: Robusta coffee production is highly sensitive to temperature. Glob. Chang. Biol..

[32] Byrareddy V, Kouadio L, Mushtaq S, Kath J, Stone R (2021). Coping with drought: Lessons learned from robusta coffee growers in Vietnam. Clim. Serv..

[33] ICO. 2021 Coffee development report. International Coffee Organization (ICO). https://www.icocoffee.org/wpcontent/uploads/2022/11/coffee-development-report-2021.pdf (2021).

[34] Coffee Board of India. Database on coffee, September 2020. Coffee Board of India. Government of India Ministry of Commerce & Industry, Bengaluru, Karnataka, India. https://www.indiacoffee.org/Database/DATABASE_Sep2020_web.pdf. Accessed 7 Nov 2022 (2020).

[35] Nath CD, Schroth G, Burslem DFRP (2016). Why do farmers plant more exotic than native trees? A case study from the Western Ghats, India. Agric. Ecosyst. Environ..

[36] CCRI. *Coffee Guide*. 231–236 (Central Coffee Research Institute (CCRI), Coffee Board of India, 2014).

[37] Abatzoglou JT, Dobrowski SZ, Parks SA, Hegewisch KC (2018). TerraClimate, a high-resolution global dataset of monthly climate and climatic water balance from 1958–2015. Sci. Data.

[38] Qin Y (2020). Agricultural risks from changing snowmelt. Nat. Clim. Chang..

[39] R Core Team. R: A language and environment for statistical computing. R Foundation for Statistical Computing, Vienna, Austria (http://www.R-project.org/) (2020).

[40] Hastie T, Tibshirani R (1987). Generalized additive models: Some applications. J. Am. Stat. Assoc..

[41] Wood SN, Pya N, Säfken B (2016). Smoothing parameter and model selection for general smooth models. J. Am. Stat. Assoc..

[42] Burnham KP, Anderson DR (2002). Model Selection and Multimodel Inference: A Practical Information-Theoretic Approach.

[43] Harrison XA (2018). A brief introduction to mixed effects modelling and multi-model inference in ecology. PeerJ.

[44] Dormann CF (2013). Collinearity: a review of methods to deal with it and a simulation study evaluating their performance. Ecography.

[45] Barton, K. MuMIn: Multi-Model Inference. R package version 1.43.17. https://CRAN.Rproject.org/package=MuMIn (2020).

[46] DaMatta FM, Ronchi CP, Maestri M, Barros RS (2007). Ecophysiology of coffee growth and production. Braz. J. Plant Physiol..

[47] Venancio LP (2020). Impact of drought associated with high temperatures on Coffea canephora plantations: A case study in Espírito Santo State, Brazil. Sci. Rep..

[48] Boreux V (2016). Agroforestry coffee production increased by native shade trees, irrigation, and liming. Agron. Sustain. Dev..

[49] Piato K (2020). Effects of shade trees on robusta coffee growth, yield and quality. A meta-analysis. Agron. Sustain. Dev..

[50] Souza HN (2010). Selection of native trees for intercropping with coffee in the Atlantic Rainforest biome. Agrofor. Syst..

[51] Cerda R (2017). Effects of shade, altitude and management on multiple ecosystem services in coffee agroecosystems. Eur. J. Agron..

[52] Mbow, C. *et al.* in *Climate Change and Land: an IPCC special report on climate change, desertification, land degradation, sustainable land management, food security, and greenhouse gas fluxes in terrestrial ecosystems* (eds P.R. Shukla *et al.*) (2019).

[53] De Leijster V (2021). Ecosystem services trajectories in coffee agroforestry in Colombia over 40 years. Ecosyst. Serv..

[54] Prado SG, Collazo JA, Irwin RE (2018). Resurgence of specialized shade coffee cultivation: Effects on pollination services and quality of coffee production. Agric. Ecosyst. Environ..

[55] Montagnon C, Cilas C, Leroy T, Yapo A, Charmetant P (2000). Genotype-location interactions for Coffea canephora yield in the Ivory Coast. Agronomie.

[56] Schipper ELF (2020). Maladaptation: When adaptation to climate change goes very wrong. One Earth.

[57] Werners SE, Wise RM, Butler JRA, Totin E, Vincent K (2021). Adaptation pathways: A review of approaches and a learning framework. Environ. Sci. Policy.

[58] Baca M, Läderach P, Haggar J, Schroth G, Ovalle O (2014). An integrated framework for assessing vulnerability to climate change and developing adaptation strategies for coffee growing families in Mesoamerica. PLOS ONE.

[59] Läderach P (2017). Climate change adaptation of coffee production in space and time. Clim. Chang..

[60] Harvey CA (2021). Transformation of coffee-growing landscapes across Latin America. A review. Agron. Sustain. Dev..

[61] Venancio LP (2019). Vegetative growth and yield of robusta coffee genotypes cultivated under different shading levels. Biosci. J..

[62] Malhi Y (2020). Climate change and ecosystems: Threats, opportunities and solutions. Philos. T. R. Soc. B.

[63] Watts M, Hutton C, Mata Guel EO, Suckall N, Peh KS-H (2022). Impacts of climate change on tropical agroforestry systems: A systematic review for identifying future research priorities. Front. For. Glob. Chang...

